# Nonfasting Apo-lipoprotein B and Triglyceride levels as a predictor of coronary heart disease in Type II diabetic patients

**DOI:** 10.12669/pjms.342.13848

**Published:** 2018

**Authors:** Nayyer-uz-Zaman, Muhammad Habeel Dar, Yasir Adnan, Lubna Noor, Gul Naz, Zahoor

**Affiliations:** 1 Dr. Nayyer- Uz- Zaman, MBBS, M.Phil Biochemistry. Department of Biochemistry, Khyber Girls Medical College (KGMC), Peshawar, Pakistan; 2 Dr. Muhammad Habeel Dar, FCPS Cardiology. Department of Cardiology, Maulvi Ameer Shah Hospital, Peshawar, Pakistan; 3 Dr. Yasir Adnan, FCPS Cardiology. Department of Cardiology, Police and Services Hospital, Peshawar, Pakistan; 4 Dr. Lubna Noor, FCPS Cardiology. Department of Cardiology, Lady Reading Hospital, Peshawar, Pakistan; 5 Dr. Gul Naz, MBBS, M.Phil Biochemistry. Department of Biochemistry, Khyber Girls Medical College (KGMC), Peshawar, Pakistan; 6 Dr. Zahoor, MBBS, Ph.D. Biochemistry. Khyber Medical College, Peshawar, Pakistan

**Keywords:** Apo-lipoprotein, Triglycerides, Acute myocardial infarction, Diabetes Mellitus

## Abstract

**Background & Objectives:**

Lipoprotein-A has been recognized as a risk factor for ischemic heart diseases. Myocardial infarction (MI) is common complication of ischemic heart disease. Diabetes play an incremental role in the development of coronary artery disease (CAD), however still there are conflicting data regarding the relationship of Lipoprotein-B and MI. We therefore wanted to evaluate the relationship of Lipo-B, MI and diabetes mellitus. Our objective was to determine the non-fasting Apo-lipoprotein B and triglycerides level among type II diabetic patients with ischemic heart disease and to compare with type II diabetic without ischemic heart disease.

**Methods:**

This was cross sectional study where two groups of patients were recruited in the study, Group-I included patient with Ischemic Heart Disease and diabetes while Group-II comprised of diabetes without Ischemic Heart Disease. Age, sex and basic demographic matching was done between the two groups. Data were collected using random sample. The comparative approach was used to see the role of diabetes in the elevation of Apo-lipoprotein B level, which is a risk factor for Ischemic heart diseases.

**Results:**

Two hundred forty eight patients (Cases: 123 Diabetic with myocardial infarction and (Control: 125 Diabetic without myocardial infarction) were included in the study. Mean Apo-B among diabetic patients with myocardial infarction was high (68.3±24.23 ng/ml) compared to non-cardiac patients (49.97±33.880 ng/ml) with a p <0.000. Marked difference was also observed in triglycerides levels where it was found very high (301.4±55.1 mg/dL) in patients of diabetes with myocardial infarction as compared to subjects without MI (137.7±84.7 mg/dL). There was positive correlation between Apo-lipoprotein and Triglycerides (P value=039).

**Conclusion:**

Based on the study result it was concluded that Apo-lipoprotein and triglycerides in diabetic patients with myocardial infarction, had higher levels compared to diabetic patients without Myocardial infarction and this could be a consequence of increase in age, insulin resistance and deficiency of insulin in the body. We also found positive correlation between Apo-lipoprotein and Triglycerides.

## INTRODUCTION

Diabetes Mellitus is one of the major risk factors of coronary heart diseases. Despite availability of effective glycemic control regimes, diabetes mellitus still remains associated with the risk of developing coronary heart diseases.^1^-^3^ Large number of clinical and research evidences indicate that both diabetic mellitus and insulin resistance lead to endothelial dysfunctions which in turn increase the risk of atherogenic function of the vascular endothelium.^1^

Hence among diabetic patients, prevention of endothelial dysfunction may be critical for preventing atherosclerosis and ischemic heart disease. Endothelial dysfunction is a major contributing factor to the pathogenesis of diabetic vascular complications.^1^ Owing to its cell regulatory function, nitric oxide is engaged in the pathogenesis of many degenerative disorders including atherosclerotic heart disease and type II diabetes.^4^ Clinical trials have identified hyperglycemia as the key determinant in the development of chronic diabetic complications. It is also evident that hyperglycemia is allied with mildly increased levels of myeloperoxidase, independent of other clinical variables. This linkage may also account for accelerated progression of the atherosclerosis in diabetic patients. Importantly Apo-lipoprotein B was found associated with almost majority of the normocholesterolemic type 2 diabetic patients.^2^

Hypertriglyceridemia is a complex clinical condition with an unclear association with atherosclerosis.^5^,^6^ Patient with moderate hypertriglyceridemia, will have high levels of low-density lipoprotein present in plasma. These smaller triglyceride-rich lipoproteins penetrate the arterial intima^7^ and appear to be preferentially trapped within the arterial wall, these residues of hyperlipidemia often lead to premature Atherosclerosis^8^,^9^ in which remnant lipoproteins play a dominant role.^10^,^11^ Therefore, increased levels of non-fasting triglycerides, reflecting increased levels of remnant lipoproteins, may predict risk of myocardial infarction (MI), ischemic heart disease (IHD), and death. Similarly, Hyper apo (B) was found in almost half of the normocholesterolemic type 2 diabetic patients. Thus, given its independent association with ischemic heart disease and that it identifies high-risk phenotypes in normocholesterolemic diabetic patients, apo(B) should be used to evaluate the lipidemic pattern of these patients^12^. Apolipoprotein B, which reflects the total mass of atherogenic particles like (VLDL, IDL, LDL) and its increase is associated with ischemic heart disease.^12^

For the correct estimation of triglycerides, studies suggest that the blood should be obtained in non-fasting state, so non-fasting blood samples be routinely used for the assessment of plasma lipid profiles. Laboratory reports should flag abnormal values on the basis of desirable concentration cut-points. Non-fasting and fasting measurements should be complementary but not mutually exclusivetime.^11^

## METHODS

This was a cross sectional descriptive study. Two groups of patients were recruited in the present study, Group-I included patients with ischemic heart disease (MI) and diabetes while Group-II included only diabetic subjects. Type 2 Diabetes was diagnosed using following criteria.

The American Diabetes Association (ADA) criteria for the diagnosis of diabetes are any of the following:^12^

- A hemoglobin A1c (HbA1c) level of 6.5% or higher; the test should be performed in a laboratory using a method that is certified by the National Glycohemoglobin Standardization Program (NGSP) and standardized or traceable to the Diabetes Control and Complications Trial (DCCT) reference assay, or
- A fasting plasma glucose (FPG) level of 126 mg/dL (7 mmol/L) or higher; fasting is defined as no caloric intake for at least 8 hours, or
- A 2-hour plasma glucose level of 200 mg/dL (11.1 mmol/L) or higher during a 75-g oral glucose tolerance test (OGTT), or
- A random plasma glucose of 200 mg/dL (11.1 mmol/L) or higher in a patient with classic symptoms of hyperglycemia (ie, polyuria, polydipsia, polyphagia, weight loss) or hyperglycemic crisis.

The comparative approach was used to see the role of diabetes in the evaluation of Apo-lipoprotein B level, which is a risk factor for IHD. The study was approved by institution ethical committee. The study was carried out in Cardiology and Endocrinology Units, Hayatabad Medical Complex (HMC), Peshawar Pakistan from December, 2015 to April, 2016. A total of 248 patients (Cases: 123 Diabetic with myocardial infarction) and (Control: 125 Diabetic without myocardial infarction) were enrolled. Randomized sampling technique was adopted to recruit the study participants. For random sampling the authors has used SPSS/ Excel. The filtered random sample then selected for further process The data were collected on predesigned Performa. It is dived into three parts; demographic, clinical examination and last part consists of biochemical profiles. After taking written informed consent a non-fasting 5 mL venous blood sample was collected from all participants while sitting. The blood was taken under aseptic techniques in sterile tube. Serum sample was stored in aliquots at 20°C and not previously thawed till batch analyzed for serum Apo B. Analysis was done within eight weeks as per their stability suggested in the protocol provided with kit followed by centrifugation for the separation of serum which was used for biochemical analysis. All biochemical parameters (TG, LDL HDL and Apo (B) were analyzed using auto-analyzer (Model No. REF 9D93,304985/R03) purchased from Abbott Pvt. Ltd. Data analysis was done using SPSS version 16. Student t-test was applied for the determination of difference between the groups for continuous biochemical parameters. Similarly simple correlation analysis (Pearson’s correlation co-efficient) was carried out to determine relationship between different variable of interest, where statistical significant will be accepted at P < 0.05.

## RESULTS

A total of 248 patients were finally included in the study out of whom 125 (50.4%) were the cases (diabetic with myocardial infarction) and 123 (49.6%) were diabetic without myocardial infarction.

The overall levels of Apo-Lipoprotein and triglyceride of both the study groups is shown in Table-I. Mean ± SD values depicts that serum Apo-B was found to be 59.1±30.8 mg/mL, TG was noted to be 218.9±149.1 mmol/L.

**Table-I:** Over all status of Apo-B and Triglycerides of Study Population (both groups).

	Apo-B mg/dL	Triglycerides mg/dL
Mean±SD	59.1±30.8	218.9±149.1
Minimum	11	20
Maximum	156	900

The results of Apo-Lipoprotein B levels were analyzed in both of the groups during this study. The results indicated that mean ± SD Apo-B among diabetic patients with myocardial infarction (patients group) was high 68.3±24.230 ng/mL as compared to non-cardiac patients (control) 49.97±33.880 ng/mL, which accounted for a mean difference of 18.341 ng/ml (CI; 10.97 - 25.70 ng/mL) indicating statistically significant difference (p = <0.000).

**Fig.1:**
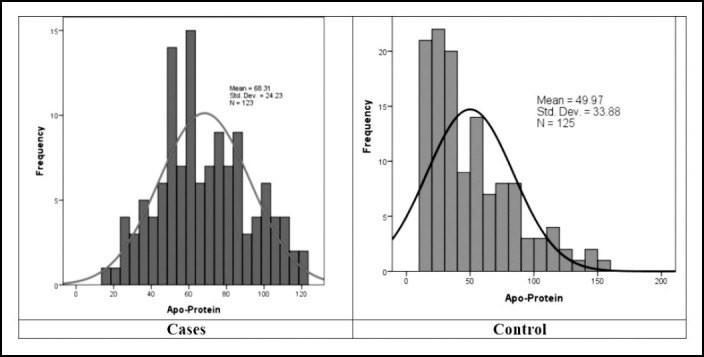
Comparative aspects of frequency distribution curve of Apo-lipoprotein among cases and control.

Apo-B level among controls showed a slight positively skewed distribution as seen in Figure -1 which indicates that majority of the participants have low Apo-B level. On other hand the Apo-B level among the patients (diabetic with MI) has more or less normal distribution which indicated that 50% participants have above the average value of Apo-B.

**Fig.2:**
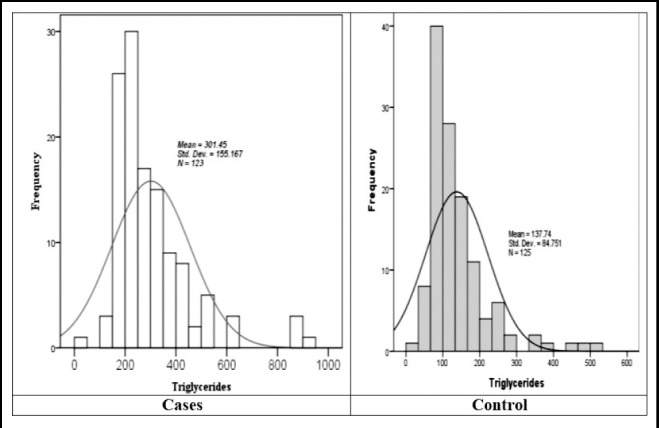
Comparative aspects of frequency distribution curve of triglyceride among cases and control.

Triglycerides (TG) were studied in the study population and the results are presented in Fig.2. Result indicate that the mean ± SD triglycerides among cases was 301.45±155.16 mg/dL (ranged from 20 to 900 mg/dL) with median of 252.00 mg/dL and mode of 217 mg/dL. Similarly, among the controls the mean ± SD triglycerides level was low as compared to case and noted to be 137.74±84.75 mg/dL (ranged 20 to 529 mg/dL) respectively. The median in this case was 110.00 mg/dL while the mode was 76 mg/dL. When we compared the two groups significant difference was observed with a p-value <0.001.

Regarding the normal curve, the graph indicates that the cases were negatively skewed due to high extreme value of triglycerides observed in the patients of diabetes with myocardial infarction, however the control had mostly skewed towards the low level indicating that they have relatively low serum triglycerides level as compared to diabetic patients with myocardial infarction.

Correlation studies were carried out separately for lipid profile of diabetic patient with and without myocardial infarction. Statistically significant positive correlation was found between Apo-lipoprotein B and triglycerides (γ = 0.039).

## DISCUSSION

The present study was designed to evaluate and test the hypothesis that very high levels of non-fasting serum triglycerides is the predictive indicator for remnant of Apo-lipoprotein B which is a risk factor for ischemic heart disease (IHD), i.e. myocardial infarction (MI). In this regard, we adopted a cross sectional analytical study to compare the *Apo-lipoprotein B level and triglycerides level among two group of patients;* Group-I included diabetic patients with IHD while Group-II included only diabetic patients without IHD patients. The comparative approach was used to see the role of diabetes in the elevation of *Apo-lipoprotein B level*, which is a risk factor for ischemic heart diseases (MI).

Our findings show an increase in plasma triglyceride levels among diabetic patients with myocardial infarction (Group-A) as compared to diabetic patients without myocardial infarction (Group-B). The present study also indicated that mean Apo-B among diabetic patients with myocardial infarction was higher 68.3±24.230 as compared to non-cardiac patients (control group) 49.97± 33.880 ng/ml, (p <0.000). However, the Apolipoprotein B (apoB) concentration reflects the number of atherogenic particles and is closely associated with atherosclerosis.^13^,^14^

The mechanism by which raised apoB and TG levels in combination increase the risk of CHD is yet uncertain. There is only one apo B molecule for each VLDL-C and LDL-C particle^15^, and because VLDL-C particles are cleared much faster than are LDL-C^16^, apo B levels in essence reflect LDL-C number, and high apo B levels reflect a relative reduction in cholesterol level. The consequence is relatively small, dense LDL-C particles derived from VLDL-C overproduction, with a reduced cholesterol /apoB ratio.^17^-^20^ In a meta-analysis, Austin (2000) reported a 60% increase in the risk of CHD decreases for every 10-Å decrease in LDL-C size. Adjustment for TGs and HDL-C reduced this to 30% increased risk, but the odds ratio remained statistically significant, demonstrating that small LDL-C is an independent risk factor.^21^ The findings of present study are in agreement with above mentioned studies (carried out elsewhere in the world).

A marked difference was also observed in triglycerides where it was found very high (301.4± 55.1) in patients with diabetes with myocardial infarction as compared to patients without MI (137.7± 84.7mg/dL). The intra individual variation in triglyceride concentrations was much higher than that of HDL-C or LDL-C cholesterol. Use of non-HDL cholesterol in the non-fasting state, although feasible, would enhance this variability. In summary, our results show that non-HDL cholesterol is a significant predictor of CVD in diabetic men and women. Since diabetic patients are at high risk for CVD morbidity and mortality, adequate risk assessment and management is imperative.

Regarding the correlation, statistically significant positive correlation was found between Apo-lipoprotein and triglycerides (γ = 0.039). We have seen skewedness among the cases with respect to most of parameters. The variation was obviously due to variation of the disease, duration of the diseases and insulin status among both of the group. Literature also indicate that insulin resistance and type II diabetes are associated with a clustering of interrelated plasma lipid and lipoprotein abnormalities, which include reduced HDL cholesterol, a predominance of small dense LDL particles, and elevated triglyceride levels. Each of these dyslipidemic features is associated with an increased risk of cardiovascular disease.^22^ Clinical trials have shown significant improvement in coronary artery disease after diabetic dyslipidemia treatment.^23^ Lipids are involved in the pathogenesis of atherosclerosis, and hence lipid profile is a basic investigation done in cases of CAD. The lipoprotein transport system is central to the mechanism by which genes, diet and hormones interact to regulate the cholesterol and triglyceride plasma levels and their tissue distribution.^24^

## CONCLUSION

Hypertriglyceridemia is a heterogeneous disorder with an unclear association with atherosclerosis. However the result of the present study indicate that higher levels of non-fasting triglycerides and Apo-B were observed in all subject regardless myocardial infarction, The marked increase of non-fasting triglycerides with associated Apo lipoprotein B indicate that these elevated non-fasting triglyceride levels were associated with increased risk of MI, IHD in men and women. This indicate that the increased levels of non-fasting triglycerides may indicate the presence of increased levels of atherogenic remnant lipoproteins.

### Authors’ Contribution

***NZ*** conceived, designed and did statistical analysis.

***MHD*** did editing of manuscript.

***YA and LN*** did data collection and manuscript writing

***GN and Z*** did review and final approval of manuscript.
